# Kratom (Mitragynine) Ingestion Requiring Naloxone Reversal

**DOI:** 10.5811/cpcem.2018.11.40588

**Published:** 2019-01-04

**Authors:** Daniel L. Overbeek, Jonathan Abraham, Brendan W. Munzer

**Affiliations:** University of Michigan, Department of Emergency Medicine, Ann Arbor, Michigan

## Abstract

Kratom (mitragynine) is a naturally occurring opioid agonist whose use has been escalating. Its suppliers advertise it as a safe alternative for opioids and a safe treatment for opioid-withdrawal symptoms. There has been controversy in the past two years regarding the legal status and lack of regulation surrounding kratom. Currently, kratom is legal and unregulated, leaving users at risk from unpredictable potencies and effects. We present the first case of successful naloxone reversal of opioid toxidrome from recreationally used kratom. We advocate further research and regulation to ensure standardized dosing to protect patients.

## INTRODUCTION

Kratom (also called mitragynine) is an opioid receptor agonist, which is a naturally-occurring compound. It is produced by *Mitragyna speciosa* and has been advertised as a safe treatment for opioid dependence as well as opiate withdrawal symptoms.^1^ Kratom is currently not a scheduled drug in the United States (U.S.) and can be legally acquired and consumed in most states.^2^ In 2016, the Drug Enforcement Administration (DEA) announced that they intended to classify kratom as a Schedule I drug; however, this decision was withdrawn after an advocacy effort by the drug’s proponents based on claims that kratom is a safe alternative to prescription opioids and for weaning opioid dependence.^3^ There have been multiple cases documenting risks of kratom.^4^,^5^ We present a unique case of kratom overdose and treatment using naloxone.

## CASE REPORT

A 38-year-old female with a history significant for depression and polysubstance abuse presented to the emergency department (ED) for altered mental status and decreased respiratory rate. She was placed in a resuscitation bay, where she was noted to be obtunded with minimal responsiveness to painful stimuli. She was also experiencing respiratory depression with bradypnea. Given clinical presentation and concern for opioid toxidrome with respiratory depression, the patient received two doses of 0.4 mg of naloxone.

Following administration, the patient’s depressed mental status resolved and respiratory rate increased. She subsequently became acutely agitated, requiring haloperidol for sedation. She was monitored in the ED, receiving supportive care and intravenous fluids. After she received haloperidol, she experienced altered mental status, which persisted for the next 12 hours. She did not have any respiratory depression during the monitoring after haloperidol administration, likely suggesting the altered mental status was related to the administration of the haloperidol.

Chart review revealed prior hospitalization for altered mental status, likely polysubstance overdose, with gas chromatography / mass spectrometry (GC/MS) during that admission positive for bupropion, venlafaxine, and kratom. During previous hospitalization, she did confirm that she had consumed kratom and otherwise denied current drug use. She was admitted to the hospital for continued altered mental status, which improved with supportive care over the next 24 hours. GC/MS analysis of her urine during this visit was positive only for the presence of kratom and did not show other opioids. The patient admitted to using kratom upon discharge, though she denied intentional overdose.

## DISCUSSION

Mitragynine is an alkaloid compound naturally produced by *Mitragyna speciosa*; it is sold as kratom in multiple different preparations.^5^,^6^ The effects vary from stimulating effects at low doses to sedating effects at high doses. Kratom has been documented to have mu-receptor agonism in humans and in vitro in mice, which would be consistent with the clinical findings in this case.^7^–^9^ Review articles currently recommend the administration of naloxone in the case of kratom overdose, mainly based on the excellent safety profile of naloxone; however, they also note that the clinical effectiveness of naloxone in reversing the effects of kratom overdose has not been proven.^10^,^11^ Prior to this case, there does not appear to have been any documented case of naloxone reversal of opioid toxidrome from kratom in humans. In this case, the patient required two doses of naloxone prior to improvement in obtundation.

One viable alternative explanation is that the patient may have consumed an opioid in addition to the kratom. We considered this to be unlikely, as the GC/MS did not identify other opioids, and the patient also endorsed kratom use and denied other drug use.

Recently there has been a surge in demand for products marketed to be an alternative for opioids as pain control and to treat opioid dependence and withdrawal. Kratom is one of the most widely known, and its use has been increasing.^12^,^13^ Recreational use has also increased, and kratom is regularly advertised as a “legal high.” Multiple online sites, including reddit.com and bluelight.org, have forums discussing its recreational use and contribute to pro-kratom advocacy.^14^,^15^ Our patient in this case had a prior history of kratom use and appeared to be using it as a recreational drug.

The increased use of kratom has been enough to draw the attention of federal agencies. In 2016, the DEA announced that they were planning to classify mitragynine as a Schedule I substance. This would have banned its sale and use in the U.S. This announcement caused an outcry from advocates for the drug, claiming that it was a safe alternative to other therapies for chronic pain. Shortly thereafter, a strong lobbying effort was undertaken to influence the DEA’s decision, and the DEA withdrew their notice shortly after the initial announcement.^3^,^16^ In November 2017, the U.S. Food and Drug Administration (FDA) released a warning against using kratom for opioid dependence and withdrawal. They identified 36 deaths associated with kratom, and refuted the claim that the drug is safe, identifying seizures and liver disease as known risks. However, the FDA did not make any statements comparing the safety of kratom to other opioids or treatments for opioid dependence.^12^,^17^

Research is not yet available to determine the relative safety of kratom for the treatment of chronic pain or opioid dependence. With the current opioid epidemic, we are in a situation where we should work to ensure the availability of safe alternatives. We call for safety studies, followed by a clear regulation of these products to ensure standardized dosing. These patients who are looking for safe alternatives are put at risk by the current unregulated plethora of products with a wide variety of dosing and preparation. The alternate is a continuation of the current system of unpredictable effects from inconsistent dosing.

**CPC-EM Capsule**

What do we already know about this clinical entity?

*Kratom (mitragynine) is a drug used as an alternative to opioids and as a complement to opioids. It has previously been shown to have opioid mu-agonist properties*.

What makes this presentation of disease reportable?

*Most toxicology resources recommend the use of naloxone to reverse kratom overdose; however, there are no previously reported cases of successful reversal with naloxone*.

What is the major learning point?

*Opioid toxidrome including respiratory depression should be treated with naloxone regardless of the specific opioid used*.

How might this improve emergency medicine practice?

*Providers should be aware of alternative opioids used by patients and the risks they face from those substances*.

## CONCLUSION

The effects of the opioid epidemic have expanded significantly in recent years. Many are looking for alternative safe treatments, and the use of kratom is also increasing. Kratom is often advertised as a safe, legal substance available as an alternative to other opioids. This case identifies the risk of kratom overdose and provides evidence that naloxone may be an effective treatment for respiratory depression from kratom use. Prior work has established the good safety profile of naloxone, and we would recommend its use in the setting of respiratory depression from kratom overdose. We call for further research and reasonable regulation of kratom to protect patients from the risks of kratom overdose.
